# Draft Genome Sequence of Mycobacterium tuberculosis Strain SNMICRO 2047-20, Isolated from Intraocular Infection

**DOI:** 10.1128/mra.00791-22

**Published:** 2022-12-21

**Authors:** Mani Vimalin Jeyalatha, Umashankar Vetrivel, Rajagopalan Harinee, Dhanurekha Lakshmipathy, Suganeswari Ganesan, Jyotirmay Biswas, Appakkudal R. Anand

**Affiliations:** a L & T Microbiology Research Centre, Vision Research Foundation, Sankara Nethralaya, Chennai, India; b ICMR-National Institute of Traditional Medicine, Indian Council of Medical Research, Belagavi, India; c Virology and Biotechnology/Bioinformatics Division, ICMR-National Institute for Research in Tuberculosis, Chennai, India; d L & T Microbiology Research Centre, Medical Research Foundation, Sankara Nethralaya, Chennai, India; e Shri Bhagwan Mahavir Vitreoretinal Services, Medical Research Foundation, Sankara Nethralaya, Chennai, India; f Department of Uveitis and Ocular Pathology, Medical Research Foundation, Sankara Nethralaya, Chennai, India; University of Maryland School of Medicine

## Abstract

Here, we communicate the draft genome sequence of an ocular Mycobacterium tuberculosis strain (SNMICRO 2047-20) that was isolated from the vitreous fluid of a patient diagnosed with endophthalmitis. The genome sequence was 4,391,538 bp long with 3,898 protein-encoding genes and clustered to the East African-Indian lineage.

## ANNOUNCEMENT

Tuberculosis (TB) is caused by Mycobacterium tuberculosis and continues to be a major public health concern (1, 2). Ocular tuberculosis (TB) is an extrapulmonary form that is challenging to diagnose due to a wide spectrum of presentations. Here, we report the coding draft genome sequences of an M. tuberculosis strain isolated from the eye. This study was approved by the institutional ethics committee of the Medical and Vision Research Foundation (854-20209-P). The M. tuberculosis strain (SNMICRO 2047-20) was grown in Middlebrook 7H9 medium using a Bactec MGIT 320 system (Becton, Dickinson) and isolated from the vitreous fluid of a 44-year female who was clinically suspected to have endogenous endophthalmitis, after 14 days of incubation. The strain was found to be sensitive to all first-line drugs tested, namely, streptomycin, isoniazid, rifampin, ethambutol, and pyrazinamide. For whole-genome sequencing (WGS), the genomic DNA was extracted using a QIAampDNA mini kit (Qiagen, Hilden, Germany). The WGS libraries were prepared using a Kapa High Throughput library preparation kit (Roche, CA), loaded onto a cBot2 system for cluster generation, and sequenced using an Illumina HiSeqXten sequencer.

The generated paired-end reads were trimmed to remove the low-quality reads and adapters using the Trimgalore 0.6.3 tool (https://www.bioinformatics.babraham.ac.uk/projects/trim_galore/). The filtered reads (mean length, 150 bp) were aligned and mapped to the Mycobacterium tuberculosis H37Rv reference genome (accession number NC_000962.3) using the SNIPPY 4.5.0 pipeline (https://github.com/tseemann/snippy) with default optimal parameters, single nucleotide polymorphism (SNP) calling, and consensus generation. Furthermore, the mapped reads were also analyzed using the Tb profiler 3.0.8 tool with default settings to infer the lineage and drug sensitivity profile. Also, the consensus-derived genomic sequences were annotated using the RAST server (3), and the coding sequence distribution was plotted using Circos software (4).

The Illumina HiSeq platform generated 992,862,146 bp of paired-end reads which on adapter trimming yielded 974,882,690 bp of high-quality reads, and the SNIPPY run generated 46 contigs with a total length of 4,391,538 bp (*N*_50_, 179,591 bp) (Table 1). The sequencing depth was found to be 221.8× with 99.4% genome coverage. On PGAP annotation of the 46 contigs (http://www.ncbi.nlm.nih.gov/genomes/static/Pipeline.html), 3,898 protein-coding genes, 51 RNA-coding genes, and 3 clustered regularly interspaced short palindromic repeat (CRISPR) arrays were predicted to span the draft genome sequence. TB profiler reported the classical regions of difference (RD) 750 deletion in this strain, and it was predicted to cluster with the East African-Indian family belonging to CAS lineage 3.

**TABLE 1:** Genomic features of Mycobacterium tuberculosis strain SNMICRO 2047-20

Parameter	Data
Lineage	3
Specimen	Vitreous aspirate
Reference-guided assembly no.	NC_000962.3
NCBI accession no.	JAHTBJ000000000
Genome size	4,391,538
No. of contigs	46
Total no. of genes	4,100
No. of protein-encoding genes	3,898
No. of CRISPR arrays	3
No. of ribosomal RNAs	3
No. of tRNAs	45
No. of noncoding RNAs	3
No. of pseudogenes	151
G+C content (%)	65.6
Genome coverage (×)	221.8
Total no. of phages	3

**FIG 1:**
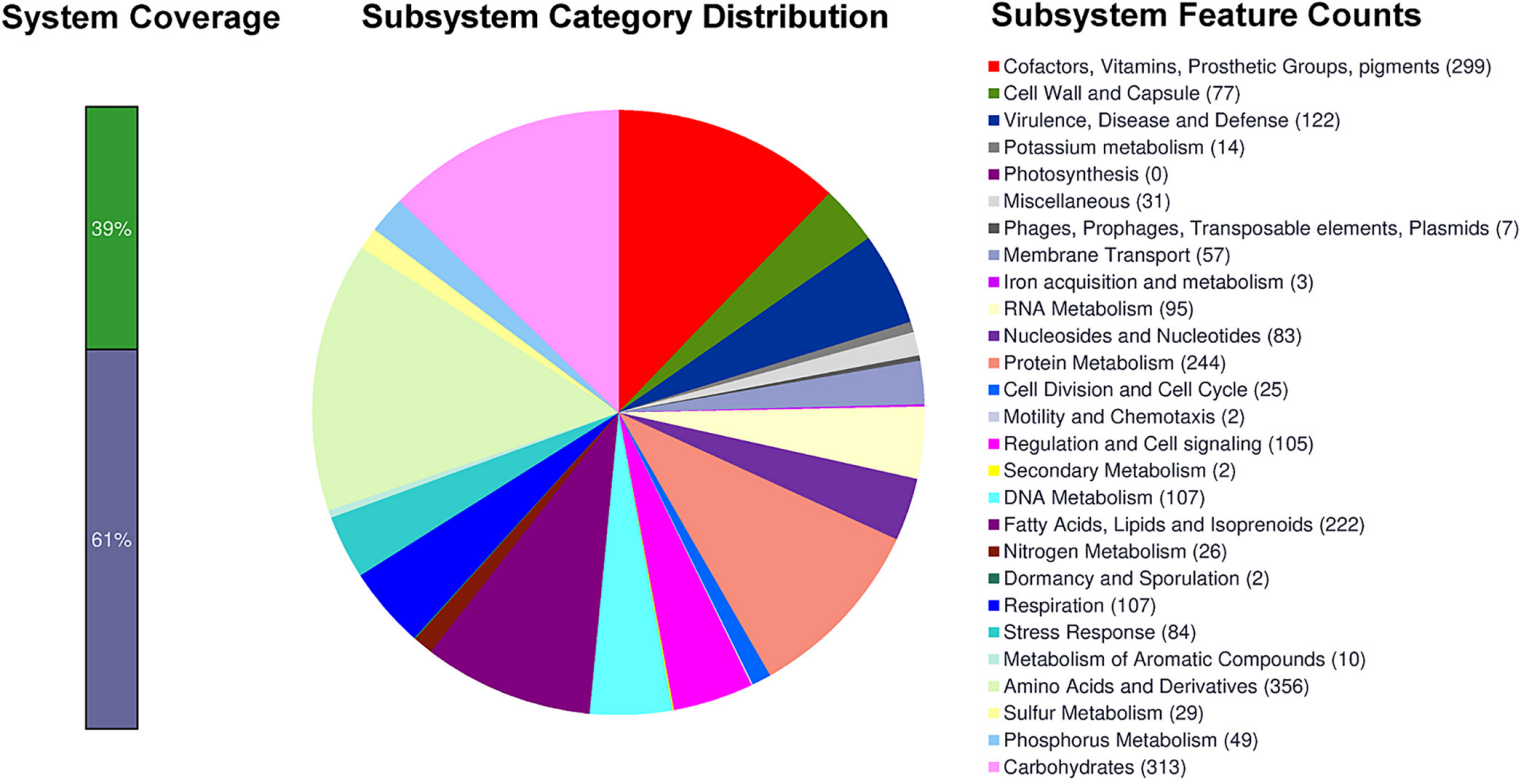
RAST annotation showing subsystem distribution of Mycobacterium tuberculosis strain SNMICRO 2047-20; a total of 405 subsystems were classified, of which 122 subsystems were confined to virulence and disease.

The RAST analysis revealed 405 subsystems (Fig. 1). A majority of the genes were involved in the synthesis of amino acids and derivatives (*n* = 356); carbohydrates (313); cofactors, vitamins, prosthetic groups, and pigments (299); protein metabolism (243); and virulence, disease, and defense (122). Notably, the strain also possesses all five T7 secretory systems (ESX 1 to 5). The draft genome sequence will provide important insights into factors involved in the evolution and adaptation of M. tuberculosis to establish infections at extrapulmonary sites, such as the eye.

### Data availability.

The draft genome sequence data of M. tuberculosis SNMICRO 2047-20 was submitted to NCBI GenBank under BioProject accession number PRJNA742522, GenBank accession number JAHTBJ000000000, and SRA accession number SRR19090886.
